# Hypothyroidism and Cardiovascular Disease: A Review

**DOI:** 10.7759/cureus.52512

**Published:** 2024-01-18

**Authors:** Diego Zúñiga, Sneha Balasubramanian, Khawar T Mehmood, Shahad Al-Baldawi, Gabriel Zúñiga Salazar

**Affiliations:** 1 Medicine, Universidad Católica de Santiago de Guayaquil, Guayaquil, ECU; 2 Internal Medicine, Madras Medical College, Chennai, IND; 3 Internal Medicine, Aster Hospital, Dubai, ARE; 4 Rheumatology, Al-Yarmouk Teaching Hospital, Baghdad, IRQ

**Keywords:** levothyroxine, endothelial dysfunction, atherosclerosis, cardiovascular risk factors, subclinical hypothyroidism, cardiovascular disease, hypothyroidism

## Abstract

Hypothyroidism is an endocrine disorder more commonly in older adults. Simultaneously, this population has an increased incidence of cardiovascular risk factors and disease, which remains the leading cause of death worldwide. Thyroid hormones (THs) promote adequate function of the cardiovascular system as they exert their effects through receptors located in the myocardium and the vasculature. In hypothyroidism, this homeostasis is disrupted, which leads to the emergence of pathogenic pathways that accelerate the progression of cardiovascular disease and aggravate its outcomes in these individuals. This article has reviewed existing literature on the relationship between hypothyroidism and cardiovascular disease (CVD). We have explored the pathogenic mechanisms linking both conditions and highlighted the prevalence of cardiovascular risk factors as well as the increased incidence of cardiovascular events in overt and subclinical diseases. Furthermore, indications of hormone replacement therapy in subclinical disease and its efficacy in reducing CVD morbidities in a particular subset of patients have been discussed.

## Introduction and background

Hypothyroidism is a common endocrine disorder characterized by a deficiency of thyroid hormones (THs), commonly as a result of insufficient hormone production or less frequently due to insufficient hormone action in target tissues [1]. The prevalence of hypothyroidism is around 4.6% in the USA, with 4.3% and 0.3% attributed to subclinical and overt disease, respectively. Hypothyroidism is six times more frequent in women and White people [2]. The incidence increases with age as it is more common in people older than 60 years, and approximately 10% of women older than this age have subclinical hypothyroidism [2].

The most common risk factor of hypothyroidism is a personal or family history of autoimmune diseases such as celiac disease or type 1 diabetes [3]. Other risk factors linked with the disease include Down and Turner syndrome, relative selenium deficiency, insufficient or excessive iodine intake, and childhood overweight [4]. Globally, iodine deficiency is still the major etiology of the disease, while in areas where iodine intake is adequate, autoimmune thyroiditis is the predominant cause, followed by iatrogenic, drugs, pituitary, and hypothalamic diseases [5]. Clinical presentation of hypothyroidism can vary from mild to potentially life-threatening signs or symptoms depending on factors such as onset of presentation, sex, age, pre-existing thyroid disease, etc. [6]. It presents most commonly with fatigue, weakness, cold intolerance, weight gain, constipation, periorbital edema, and dry and coarse skin [7].

Due to the lack of specificity of these manifestations, diagnosis is made by laboratory measurements of serum-free thyroxine (T4) and thyrotropin-stimulating hormone (TSH) levels [6]. Increased TSH levels indicate hypothyroidism, while free T4 can distinguish between overt and subclinical disease, presenting with low and normal levels, respectively [3]. Levothyroxine is the treatment of choice because of its efficacy in normalizing TSH, relieving signs and symptoms, and its minimal adverse effects [6]. Untreated or inadequate hormone therapy has negative consequences in various organs, being the cardiovascular one of the most affected systems and a major cause of mortality in these patients [8]. The role of THs in the normal functioning of the heart and vascular physiology, and how alterations in their levels impact cardiovascular function, is something that research has particularly focused on [8,9]. Hypothyroidism results in decreased cardiac output, impaired left ventricular function, and increased vascular resistance [10]. Furthermore, studies have shown a higher prevalence of cardiovascular risk factors with both subclinical and overt hypothyroidism, such as dyslipidemia and hypertension as well as endothelial dysfunction, all of which increase the risk of progression to cardiovascular disease (CVD), most of which are discussed in this article [11]. This review article aims to explore the likely mechanisms that link hypothyroidism with cardiovascular disease, highlight the prevalence of cardiovascular risk factors and the incidence of CVD outcomes in individuals with thyroid dysfunction, and discuss the effectiveness of thyroid hormone replacement therapy in the management of cardiovascular disease comorbidities in these patients.

## Review

Mechanisms linking hypothyroidism with CVD 

Thyroid hormones play an important role in the homeostasis of the cardiovascular system, as TH receptors are present in both vascular endothelium and myocardium [11,12]. It has been observed that even subtle alterations of the serum TH levels have a negative effect on the cardiovascular system, associated with increased risk of CVD and all-cause mortality [13,14]. The two main mechanisms that link hypothyroidism with CVD are accelerated atherosclerosis formation and myocardial dysfunction [11].

Low levels of TH increase the rate and extension of atherosclerosis through multiple mechanisms, such as endothelial dysfunction, abnormal lipid metabolism, changes in blood pressure, hemostatic abnormalities, and insulin resistance [9]. Endothelial dysfunction occurs when the balance between vasoconstriction and vasodilation is disturbed [15]. The main substance that mediates endothelial function and vasodilation is nitric oxide (NO) [15]. Sheer stress is the main stimulant for NO release from the vascular endothelial tissue, but not the only one [15]. Thyroid hormones can also stimulate endothelial NO production through nongenomic actions by activating the phosphatidylinositol 3-kinase (PI3K) and the serine/threonine-protein kinase signaling pathways [16,17]. Low levels of NO production in individuals with hypothyroidism will eventually lead to endothelial dysfunction, which has impacts beyond vascular tone modulation [15]. The endothelium also has anti-inflammatory and anti-atherogenic functions, as it decreases the expression of cytokines, the endothelial permeability, and the adhesion of platelets [15,16]. Endothelial dysfunction caused by reduced NO levels results in decreased flow-mediated dilation and a series of actions that contribute to the formation of atherosclerosis, such as platelets and monocyte adhesion, low-density lipoprotein (LDL) oxidation, expression of thrombogenic factors and migration and proliferation of smooth muscle cells [15].

Abnormal lipid metabolism occurs in patients with deficient levels of TH as they participate in its regulation via various mechanisms [10]. Thyroid hormones stimulate the gene expression of both the hepatic hydroxymethylglutaryl coenzyme A reductase (HMG-CoA) enzyme and the low-density lipoprotein receptor (LDL-R) [16,17]. When there are low levels of THs, cholesterol synthesis in the liver exceeds the clearance of LDL-cholesterol (LDL-C, resulting in elevated LDL levels) [17]. Along with hypercholesterolemia, increased levels of triglycerides (TGs) are commonly observed in this condition, as thyroid hormones promote adequate activity of the lipoprotein lipase, whose function is to degrade TGs in circulating chylomicrons [18]. Beyond the negative ratio of LDL-C to high-density lipoprotein (HDL)-C, oxidation of LDL is also increased in this disease, which strongly promotes atherogenesis [15].

Alterations in the vasculature in individuals with hypothyroidism could lead to changes in blood pressure, the thickness of intima-media layers of main vessels, and arterial stiffness, all being surrogates for atherosclerosis development [11,19]. The diastolic component of the blood pressure is predominantly elevated in patients with low levels of TH due to increased systemic vascular resistance (SVR) caused mainly by reduced expression of endothelial (NO), as mentioned earlier [19]. Additionally, hemostatic abnormalities such as antithrombin activity, fibrinolysis reduction, and increased levels of factor VII and fibrinogen have been shown in these patients, making them susceptible to thromboembolic events [11,20].

**Figure 1:**
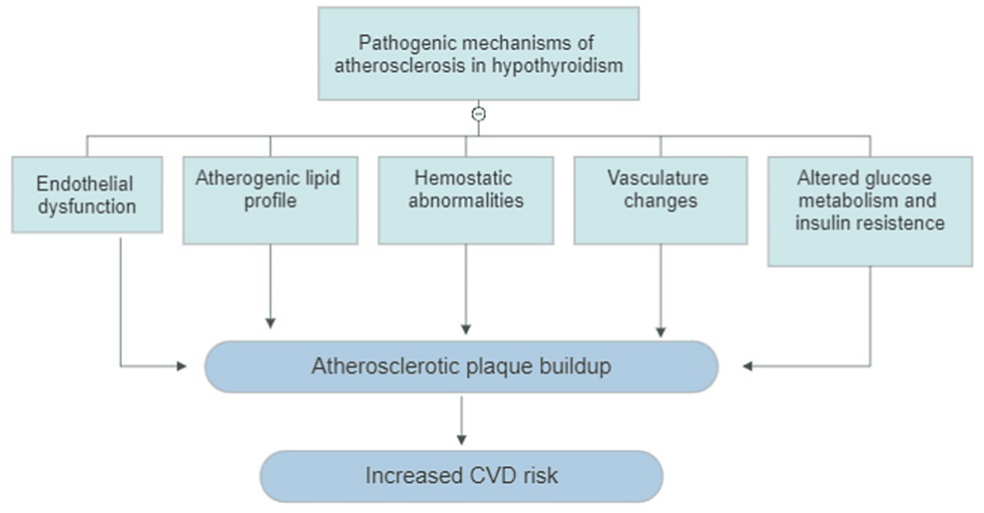
Summary of the pathogenic mechanisms inducing and accelerating atherosclerotic plaque formation in hypothyroidism CVD - cardiovascular disease Image credit: Gabriel Zúñiga Salazar

Serum TSH has a positive correlation with insulin resistance and hyperglycemia. The underlying mechanism behind insulin resistance in hypothyroidism might involve increased serum-free fatty acids, disruptions in leptin's effects at the hypothalamus, and a compromised translocation of the glucose transporter type 4 (GLUT4) in peripheral tissues [21]. In addition, TSH promotes the expression of glucose 6-phosphate and phosphoenolpyruvate carboxins (PEPCK) in hepatocytes, leading to increased glucose production in the liver [22]. The summary of the pathogenic mechanism that promotes atherosclerotic plaque buildup in hypothyroidism is shown in Figure 1. 

Functional myocardial changes are the second mechanism that could potentially link hypothyroidism with cardiovascular disease [9]. The effects of THs on the myocardium are mediated through genomic and nongenomic actions [17]. Genomic actions of the thyroid hormones in the cardiac myocytes are exerted through the TH nuclear receptors located intracellularly [17]. These receptors have an increased affinity for T3 than T4, and the binding regulates positive and negative cardiac gene expression [23]. The alpha myosin heavy chains (a-MHC) and the sarcoplasmic reticulum calcium adenosine triphosphatase (SERCA2) are upregulated, favoring cardiac contractility. At the same time, its counterpart, the phospholamban (PLB), is downregulated, promoting the release and reuptake of calcium ions in cardiac myocytes, which improves ventricular relaxation [23-25]. This lusitropic effect is a distinctive feature of thyroid hormones on cardiac contractility, as individuals with hypothyroidism have been observed to have a compromise in diastolic function due to reduced calcium ions cycling [11,26]. The inotropic effect is directly mediated via the upregulation of the β1­-adrenergic receptor gene expression of the β1­-adrenergic receptor, while the chronotropic effect is mediated via combined genomic and nongenomic mechanisms involving ions channels and the expression of the β1­-adrenergic receptors [17,26]. Overall, thyroid hormone deficiency causes myocardial dysfunction by depressing the left ventricular diastolic and systolic dysfunction at rest and during exercise respectively, worsening the cardiovascular performance in older individuals or with pre-existing cardiac conditions [9]. 

CVD comorbidities in patients with thyroid dysfunction 

The relationship between hypothyroidism and cardiovascular events is something that has been studied for decades since the first autopsies of individuals with myxedema reported diffuse atherosclerosis [27]. Since then, different studies have shown an increased risk for cardiovascular diseases such as myocardial infarction, cerebrovascular diseases, and heart failure in poorly controlled hypothyroid patients [12]. The underlying mechanisms mentioned before explain how well-known risk factors for cardiovascular disease could be possibly precipitated and exacerbated in patients with thyroid dysfunction [12]. More recent studies have demonstrated a higher prevalence of established risk factors in patients with overt and subclinical hypothyroidism, such as dyslipidemia, hypertension, diabetes, and metabolic syndrome [18,28]. 

Prevalence of CVD Risk Factors

Dyslipidemia is the most common finding in individuals with overactive thyroid dysfunction, with studies in lipid profile showing that 90% of patients with overt hypothyroidism have hyperlipidemia, in particular, an increase of serum total cholesterol and LDL-C [29]. However, evidence of dyslipidemias in subclinical diseases is contradictory. Before the 2000s, NHANES and Whickham surveys, both major population-based studies, reported no association between hyperlipidemia and subclinical hypothyroidism [30,31]. The same findings have been reported in more recent studies [32,33]. A prospective population-based cohort study was conducted in Iran over a period of one year in a sample population of 5154 aged between 25-55 years who were randomly selected [33]. It was found that the lipid profile was not significantly different between the subclinical hypothyroid and control group [33]. In contrast, a meta-analysis conducted by Treister-Goltzman et al. in 2018, which included 35 case-control and cohort studies evaluating lipid profiles in patients with mild subclinical hypothyroidism compared to euthyroid, showed that LDL-C and TG were significantly higher and that HDL-C was significantly lower, concluding that subclinical disease is associated with an increase in atherogenic lipoproteins [34].

Hypertension is a well-known risk factor for negative cardiovascular outcomes, including mortality from coronary artery and cerebrovascular diseases [35]. Studies have shown that the lifetime risk of developing cardiovascular disease in patients with hypertension is 20% more than in normotensive patients [36]. Hypothyroidism is mostly associated with diastolic hypertension or an isolated increase of diastolic components [37,38]. However, some studies have shown both systolic and diastolic hypertension in hypothyroid patients [39,40]. A population-based health survey study was conducted in Norway with a sample population of 5872 subjects aged between 30 and 75 years [39]. It was found that both systolic and diastolic blood pressure was higher, 4.0 mm and 2.7 mm Hg, respectively, in individuals with higher TSH levels compared to normal levels, thereby concluding that there was a significant, even if modest, positive association between serum TSH levels and blood pressure [39]. In addition, changes in blood pressure have also been reported in patients with subclinical hypothyroidism [41]. In a cross-sectional survey study conducted by Liu et al. in China in a sample population of 1319 subjects aged 18-85 years divided into subclinical hypothyroidism and euthyroid [41]. It was found that the prevalence of hypertension in patients with subclinical hypothyroidism was significantly higher than in euthyroid patients (41.3 vs. 25.6%, p<0.05), thus concluding that subclinical hypothyroidism could also increase the prevalence risk of hypertension [41].

Diabetes is another major risk factor for the development of cardiovascular diseases, and it is associated particularly with unfavorable outcomes, even so, that the all-cause mortality risk associated with this disease is comparable to the all-cause mortality risk associated with previous myocardial infarction [36,42,43]. The risk of diabetes and its spectrum, including hyperglycemia, insulin resistance, and hyperinsulinemia, is increased in patients with thyroid dysfunction [44,45]. A descriptive cross-sectional study was conducted by Poudel et al. in Nepal in 2021 with a sample of a total of 520 patients with overt primary hypothyroidism. It was found that the prevalence of diabetes out of all 520 patients was 203 (39.04%), with a higher proportion of females than males, thus showing a superior prevalence of diabetes in these patients than any other similar studies [46]. A meta-analysis study conducted by Roa Dueñas et al. in 2022 included seven prospective studies evaluating the association of thyroid disorders and type 2 diabetes. It was found that the pooled hazard ratio in individuals with hypothyroidism was 1.26 (95% CI, 1.05-1.52) for the risk of type 2 diabetes, submitting evidence of the increased risk for diabetes in hypothyroidism [47].

Metabolic syndrome consists of the following four metabolic abnormalities criteria: hypertension, hyperglycemia, abdominal obesity, and dyslipidemia, specifically low HDL and high TG levels [48,49]. Thresholds and numbers of criteria needed for diagnosis of metabolic syndrome vary along guidelines, with the National Cholesterol Education Program (NCEP) Adult Treatment Panel III (ATP III) being universally used. Three of the five parameters mentioned above are required for diagnosis [48]. Metabolic syndrome is a well-established risk factor for the development of both cardiovascular disease and type 2 diabetes [49,50]. Meta-analysis studies have reported individuals with metabolic syndrome have a risk between 1.53 to 2.18 for developing cardiovascular diseases and a 10-fold increased risk for diabetes compared to individuals without the syndrome [50-52]. In hypothyroidism, there is an increased prevalence of the distinct parameters of metabolic syndrome, but the prevalence of having three of the five criteria needed for the diagnosis is contradictory [53,54]. A meta-analysis conducted by Eftekharzadeh et al. in 2015 included eight studies evaluating the odds ratio of metabolic syndrome in patients with subclinical hypothyroidism [55]. Even though, in individuals with subclinical hypothyroidism, central obesity was significantly prevalent (OR=1.43, 95% CI 1.04-1.96), there was no significant difference in the prevalence of metabolic syndrome compared to euthyroid individuals (OR=1.13, 95% CI 0.95-1.34) [55]. The above study can be contrasted with another meta-analysis study done in 2020, which included 19 studies evaluating the association between subclinical hypothyroidism (SCH) and metabolic syndrome, which found a pooled odds ratio of 1.28 (95% CI: 1.19 to 1.39, p=0.04) for metabolic syndrome in these patients compared to euthyroid individuals [55,56]. These significant associations of hypothyroidism with the above CVD risk factors are shown in Table 1. 

**Table 1:** Summary of studies showing associations between hypothyroidism and cardiovascular risk factors HT - hypothyroidism; SHT - subclinical hypothyroidism

References	Design	Population sample	Population characteristics	Variable	Findings
Alamdari et al. (2015) [33]	Cohort study	288 SHT patients, 4766 healthy controls	Adults between 25-75 years in Iran	Dyslipidemia	Lipid profile did not differ between subclinical hypothyroid and control groups.
Treister-Goltzman et al. (2021) [34]	Meta-analysis	SHT patients and healthy controls	Adults	Dyslipidemia	LDL-C and TG were significantly higher and HDL-C was significantly lower in subclinical disease compared to control
Liu et al. (2010) [41]	Cross-sectional study	93 SHT patients, 1266 healthy controls	Adults aged 18-85 years in China	Hypertension	Prevalence in subclinical hypothyroidism was 41.3%. Prevalence in 25,6% in controls
Poudel et al. (2023) [46]	Cross-sectional study	520 Overt HT patients	Adults over 18 in Nepal	Diabetes	Prevalence 39.04%
Ding et al. (2021) [56]	Meta-analysis study	SHT patients and healthy controls	Adults	Metabolic syndrome	The pooled OR in subclinical hypothyroidism was 1.28 (95% CI: 1.19 to 1.39) compared to euthyroid patients

Incidence of CDV Outcomes

There is no standard definition of cardiovascular diseases, and usually, different entities are involved in this terminology depending on the author [57]. The American College of Cardiology/American Heart Association (ACC/AHA) defines cardiovascular diseases in relation to the principal pathologic process, atherosclerosis, as atherosclerotic cardiovascular disease (ASCVD) [58]. Four are the main disease groups specific to this terminology: coronary heart disease (CHD), cerebrovascular disease, peripheral artery disease, and aortic atherosclerosis disease [58]. It has been reported that overt hypothyroidism is associated with an increase in cardiovascular disease's morbimortality of 20% to 80%, in particular CHD and cerebrovascular diseases [16,59]. 

Stable angina, acute coronary syndrome (ACS), and/or heart failure are manifestations of CHD [58]. It has been known that CHD is the most common ASCVD observed in patients with thyroid dysfunction [16]. A cross-sectional study was conducted over a period between 2013 and 2018 in Saudi Arabia in a sample population of 412 adult hypothyroid patients [60]. CHD was diagnosed in 22% of the patients, and it was also noted that TSH levels were significantly higher in patients with CHD than patients without, with increased odds of presenting coronary artery disease (CAD) by 4.8% for every 1 mIU/L increase in serum TSH level [60]. Subclinical disease also is associated with an increased risk of CHD, even though it is not associated with mortality from cardiovascular causes [61]. In a meta-analysis study conducted by Singh et al. that included six studies evaluating the association between subclinical hypothyroidism and coronary heart disease, it was found significantly higher risk of CHD (RR=1.5) than control groups [61].

Low levels of THs can negatively affect myocardial morphology and physiology, largely compromising diastolic function [8]. Nevertheless, the degree of dysfunction has not been associated with developing heart failure but instead exacerbates and worsens this condition [62]. A historical cohort study was conducted by Ro et al. over a period of 25 years with a sample of 52,856 patients aged >18 at the time of baseline serum TSH measurement [63]. It was found that individuals with hypothyroidism and congestive heart failure (CHF) had a significantly higher risk of hospitalization (HR: 1.86) due to CHF exacerbation than in the euthyroid group [63]. In addition, a prospective cohort was conducted by Kannan et al. in the USA with a sample of 1365 patients with pre-existing heart failure to assess the prevalence of thyroid dysfunction and its correlation with cardiovascular outcomes [64]. It was found that higher levels of TSH were associated with severe heart failure and that hypothyroidism was associated with an increase in heart transplantation, ventricular assist device placement, and mortality than euthyroid patients [64]. The authors concluded that in patients with pre-existing HF, hypothyroidism is associated with poor prognosis [64].

Hypothyroidism is speculated to elevate the risk of cerebrovascular diseases, such as stroke and transient ischemic attack, through atherogenic alterations linked with low TH levels [56,65]. However, conflicting results of this direct correlation have been found [66,67]. In a systematic review/meta-analysis study conducted by Chaker et al., which included a sample of six cohort studies evaluating the risk for stroke in patients with subclinical thyroid dysfunction, it was found no significant difference between the stroke risk in this group compared to the euthyroid group (HR =1.08, 95% CI 0.87-1.34) [66]. On the other hand, a study was conducted by Yang et al. in Taiwan with a sample of 5793 hypothyroidism patients with corresponding control subjects [67]. It was found that hypothyroidism increased 89% of the risk of developing follow-up stroke by concluding that this condition is comparable to established risk factors, such as diabetes, hypertension, etc., for cerebrovascular diseases [67]. These significant associations between hypothyroidism and the above CVD risk events are shown in Table 2. 

**Table 2:** Summary of studies showing associations between hypothyroidism and risk of cardiovascular disease HT - hypothyroidism; SHT - subclinical hypothyroidism; CAD - coronary artery disease; CHF - congestive heart failure; CHD - coronary heart disease

References	Design	Sample population	Population characteristics	Variable	Findings
Mahzari et al. (2022) [60]	Cross-sectional study	412 overt HT patients	Adults in Saudi Arabia	CAD	Prevalence of 21.8% with CHD. The mean of TSH was significantly higher in CAD patients than non-CAD patients before and at diagnosis with CAD (p<0.001)
Singh et al. (2008) [61]	Meta-analysis study	SHT patients and healthy controls	Adults	CAD	The risk of CAD in subclinical hypothyroidism was found higher (RR=1.5) than in control groups.
Ro et al. (2018) [63]	Cohort study	3065 overt HT patients, 49,791 healthy controls	Adults with congestive heart failure (CHF)	Hospitalization due to exacerbation of CHF	Hypothyroidism was associated with a higher risk of hospitalization in those with CHF (adjusted hazard ratio = 1.86, CI 1.17-2.94) compared to euthyroid.
Chaker et al. (2014) [3]	Meta-analysis study	SHT patients and healthy controls	Adults	Stroke	No significant difference in stroke risk between both groups (HR =1.08 (95 % CI 0.87-1.34)
Yang et al. (2015) [67]	Case-control study	5793 overt HT patients, 5793 healthy controls	Adults in Taiwan	Stroke	Hypothyroidism increased 89% of the hazard of developing follow-up CVD (adjusted HR, 1.89) compared to euthyroid.

Therapy replacement therapy in hypothyroidism: efficacy on CVD comorbidities 

While there is established evidence that replacement therapy with levothyroxine in overt hypothyroidism significantly improves cardiovascular risk factors and CVD outcomes, the evidence showing benefit in reducing CVD comorbidities in subclinical disease is not clear [68]. Based on available data, guidelines from the American Thyroid Association recommend treating subclinical hypothyroidism when TSH levels are above 10 mU/L, as is at this point where CVD risk is comparable to overt disease [68]. However, it seems that other factors besides TSH levels, such as age, CVD risk, and other comorbidities, may also influence the CVD outcomes with levothyroxine, thereby making the decision to treat or not in subclinical disease unclear [69]. 

Studies evaluating the effect of levothyroxine in subclinical hypothyroidism in CVD risk factors, in particular dyslipidemias, have shown heterogeneous results [69]. A randomized placebo-controlled study, which included subclinical hypothyroid and euthyroid patients, evaluated the response in lipid profile after six months of levothyroxine vs. placebo treatment [70]. It was found that hormone replacement therapy resulted in a significant decrease in both total cholesterol and LDL-C concentrations (p=0.003), and no changes occurred in the placebo group [70]. A similar study conducted by Monzani et al. showed only a significant decrease in LDL-C and total cholesterol when baseline TSH levels were above 10 mU/L [71]. In addition, numerous studies have reported improvement of cardiovascular risk surrogates, such as carotid intima-media thickness (IMT) and endothelial function, subsequent to thyroxine treatment in patients with subclinical hypothyroidism [72-74].

While results from small studies show the benefits of treatment for subclinical disease in improving cardiovascular disease risk factors and markers, it remains uncertain whether these risk reductions would eventually result in benefits concerning the incidence of CVD outcomes [75]. Evidence associating reduction of CVD events in these patients treated with hormone replacement therapy is limited but significant in the younger population (<75 years) with high risk of CVD (diabetes, hypertension, dyslipidemia) according to few studies [16,76]. A retrospective cohort study was conducted by Razvi et al. in the United Kingdom, with a sample of 4735 subjects with subclinical hypothyroidism evaluating the effect of levothyroxine on CVD outcomes [16]. It was found that only in young patients (<70 years) there were less ischemic heart disease events (HR 0.61, 95% CI 0.39-0.95) and mortality due to circulatory diseases (HR 0.54, 95% CI 0.37-0.92) [16]. Another cohort study evaluating the effects of levothyroxine treatment in subclinical hypothyroid patients on CVD outcomes, including myocardial infarction, CVD deaths, and all-cause mortality, showed no benefits except in patients aged less than 65 [76]. The above studies can be contrasted with a randomized controlled clinical trial study done in adults older than 65 years with subclinical hypothyroidism, comparing the CVD in the levothyroxine vs placebo group [77]. It was found that fatal or nonfatal cardiovascular events and all-cause mortality were not significantly different between both groups [77]. These effects of levothyroxine in cardiovascular comorbidities in patients with subclinical hypothyroidism are shown in Table 3. 

**Table 3:** Summary of studies showing the effect on cardiovascular risk factors and outcomes of levothyroxine treatment in subclinical hypothyroidism HT - hypothyroidism; SHT - subclinical hypothyroidism; TC - total cholesterol; LDL-C - low-density lipoprotein cholesterol; MI - myocardial infarction

References	Design	Sample population	Population characteristics	Variable	Findings
Caraccio et al. (2002) [70]	Randomized placebo-controlled study	49 SHT patients, 33 healthy controls	Adults in Italy	Lipid profile	Levothyroxine treatment resulted in a significant decrease of both TC and LDLc concentrations (p=0.003)
Monzani et al. (2004) [71]	Double-blind, placebo-controlled study	45 SHT patients, 32 healthy controls	Adults in Italy	Lipid profile	Levothyroxine replacement significantly reduced both total and LDL cholesterol (p<0.0001) only when TSH levels were above 10mU/L.
Razvi et al. (2012) [73]	Retrospective cohort-Study	2453 levothyroxine-treated SHT patients, 2282 untreated SHT	Adults >40 years old in UK	Ischemic heart disease	Treatment of subclinical hypothyroidism with levothyroxine was associated with fewer ischemic heart events in younger individuals (<70 years) (multivariate-adjusted HR, 0.61; 95% CI, 0.39-0.95).
Andersen et al. (2015) [76]	Retrospective cohort study	2483 levothyroxine-treated SHT patients, 9729 untreated SHT	Adults >18 years old in Denmark	MI, cardiovascular death and all-cause mortality	Beneficial effects were found in levothyroxine-treated patients on MI, cardiovascular death, or all-cause mortality, only in patients under the age of 65 years (IRR 0.63, 95% CI: 0.40 to 0.99).
Stott et al. (2017) [77]	Double-blind, randomized, placebo-controlled trial	369 levothyroxine-treated SHT patients, 369 untreated SHT	Adults	Fatal or nonfatal cardiovascular events	Fatal or nonfatal cardiovascular events and all-cause mortality were not significantly different between both groups.

Limitations 

This review focuses primarily on the effect of hypothyroidism in the development of cardiovascular comorbidities and does not review the multifactorial etiology of this condition. Similarly, this paper does not discuss other factors that play a role in CVD outcomes when evaluating the efficacy of hormone replacement therapy in hypothyroidism.

## Conclusions

Thyroid hormones play a crucial role in maintaining homeostasis within major organ systems, with the cardiovascular system particularly affected in cases of thyroid dysfunction. This review delves into the interconnected pathways through which hypothyroidism and cardiovascular events are linked. As highlighted by the research covered here, well-established cardiovascular risk factors like hypertension, atherogenic dyslipidemia, and insulin resistance are prevalent in individuals with both overt and subclinical hypothyroidism. Additionally, there is an elevated likelihood of cardiovascular incidents such as coronary heart disease and strokes, with worse outcomes in patients suffering from this condition when compared to those with normal thyroid function. The use of hormone replacement therapy is nearly always recommended for individuals with overt hypothyroidism, but the indications for subclinical disease are more limited. Studies showing benefits in CVD risk and outcomes of levothyroxine treatment in a particular subset of patients with subclinical disease have been discussed.

In summary, the clinical significance of this article is to expose hypothyroidism as an independent mediator in initiating and exacerbating cardiovascular disease in both overt and subclinical presentations. In light of these considerations, it becomes important to screen all hypothyroid patients for CVD risk factors and to initiate early treatment to prevent cardiovascular complications. In addition, screening for thyroid disease in patients with CVD should be considered as benefits from hormone replacement therapy have shown promising results. It might be challenging to know where to start treatment when it comes to subclinical disease as there is limited data on its benefits. Finally, we recommend more important studies to be performed comparing CVD comorbidities risk between overt and subclinical hypothyroidism with adjusted variables and studies evaluating the efficacy of hormone replacement treatment in subclinical disease of patients with different TSH levels.
